# The genome of the diatom *Chaetoceros tenuissimus* carries an ancient integrated fragment of an extant virus

**DOI:** 10.1038/s41598-021-00565-3

**Published:** 2021-11-24

**Authors:** Yuki Hongo, Kei Kimura, Yoshihiro Takaki, Yukari Yoshida, Shuichiro Baba, Genta Kobayashi, Keizo Nagasaki, Takeshi Hano, Yuji Tomaru

**Affiliations:** 1 Fisheries Resources Institute, Japan Fisheries Research and Education Agency, 2-12-4 Fukuura, Kanazawa, Yokohama, Kanagawa 236-8648 Japan; 2 Faculty of Agriculture, Saga University, 1 Honjo-machi, Saga, 840-8502 Japan; 3 Super-Cutting-Edge Grand and Advanced Research Program, Japan Agency for Marine-Earth Science and Technology, 2-15 Natsushima-cho, Yokosuka, Kanagawa 237-0061 Japan; 4 United Graduate School of Agricultural Sciences, Kagoshima University, 1-21-24 Korimoto, Kagoshima, 890-0065 Japan; 5 Faculty of Science and Technology, Kochi University, 200 Otsu, Monobe, Nankoku, Kochi 783-8502 Japan; 6 Fisheries Technology Institute, Japan Fisheries Research and Education Agency, 2-17-5 Maruishi, Hatsukaichi, Hiroshima 739-0452 Japan

**Keywords:** Eukaryote, Virus-host interactions, Coevolution, Genome

## Abstract

Diatoms are one of the most prominent oceanic primary producers and are now recognized to be distributed throughout the world. They maintain their population despite predators, infections, and unfavourable environmental conditions. One of the smallest diatoms, *Chaetoceros tenuissimus*, can coexist with infectious viruses during blooms. To further understand this relationship, we sequenced the *C. tenuissimus* strain NIES-3715 genome. A gene fragment of a replication-associated gene from the infectious ssDNA virus (designated endogenous virus-like fragment, EVLF) was found to be integrated into each 41 Mb of haploid assembly. In addition, the EVLF was transcriptionally active and conserved in nine other *C. tenuissimus* strains from different geographical areas, although the primary structures of their proteins varied. The phylogenetic tree further suggested that the EVLF was acquired by the ancestor of *C. tenuissimus*. Additionally, retrotransposon genes possessing a reverse transcriptase function were more abundant in *C. tenuissimus* than in *Thalassiosira pseudonana* and *Phaeodactylum tricornutum*. Moreover, a target site duplication, a hallmark for long interspersed nuclear element retrotransposons, flanked the EVLF. Therefore, the EVLF was likely integrated by a retrotransposon during viral infection. The present study provides further insights into the diatom-virus evolutionary relationship.

## Introduction

Diatoms (Bacillariophyta) are an important group of oceanic eukaryotic phytoplankton accounting for approximately 40% of primary marine production^1,2^. The TARA Oceans project, a global plankton sampling campaign, has highlighted the significance of diatoms in global biogeochemical cycles^3,4^. Research on the dynamics of diatoms is, therefore, important to understand global ecosystems. Generally, the growth and photosynthetic activity of diatoms are higher than those of other phytoplankton and are often responsible for the blooms found in coastal and upwelling regions, thus playing a role as a major food resource for zooplankton, larvae, and filter feeders^5,6^ among others. Diatom dynamics, in addition to predation, are primarily controlled by environmental factors, such as water temperature, light, and nutrients^5,7^. Other than these abiotic factors, diatom populations are exposed to diverse biological stressors. Over the past three decades, numerous studies have suggested that viral infections are a major determinant of phytoplankton fate in aquatic environments^8^. Indeed, knowledge regarding diatom viruses has accumulated rapidly since their first report in 2004. Two main virus groups infecting diatoms have been identified, namely single-stranded (ss) DNA and ssRNA diatom viruses^9^. Diatom cell death due to viral infections can be readily observed in the laboratory, with infected cultures dying off in a few days^9^. In nature, however, the diatom population does not exhibit a rapid decrease in numbers, even in the presence of these infectious viruses; therefore, they seem to have the ability to coexist in the same region^10,11^. Hence, diatoms are thought to have evolved a mechanism to resist viral infections, as has been observed in other phytoplankton-virus systems.

The virus resistance mechanisms of eukaryotic microalgae have been reported by numerous researchers, e.g., variation in susceptibility in the host cell^12–14^, blockade of intracellular virus genome replication^15^, bacterially mediated virus resistance^16^, and host cell physiological barriers^17,18^. In addition, recent genomic and transcriptomic analyses have revealed virus resistance systems at higher resolutions. For example, variabilities in genomic islands containing the viral-attachment genes of the cyanobacteria *Prochlorococcus* facilitate host-virus coexistence^19^. Moreover, for the smallest eukaryotic phytoplankton, *Ostreococcus tauri* (Mamiellophyceae), the size of the hypervariable region in chromosome 19 differs greatly among strains, which is assumed to be closely related to its susceptibility to infection by its dsDNA virus, OtV^20,21^. Furthermore, in many hosts organism-virus relationships, whole- or partial- viral genome sequences present in the host DNA can act as a viral resistance factor, e.g., superinfection exclusion and RNA interference related mechanisms^22–25^. To gain a more in-depth understanding of the host-virus systems, recent studies have highlighted the importance of using host genome analyses^26–28^.

The marine planktonic diatom, *Chaetoceros tenuissimus* Meunier (Bacillariophyta, Centrales), is rectangular in the girdle view and is one of the smallest (~ 5 µm) diatoms. This species is widely distributed and is observed in Japanese coastal waters^10^, the Narraganset Bay^29^, the Mediterranean Sea^30,31^, and the San Matıas Gulf^32^. A previous study showed that *C. tenuissimus* has a high growth rate of at least three divisions per day and blooms during spring and autumn to levels of ~ 10^7^ cells/l^10^. To date, four different viruses capable of infecting *C. tenuissimus* have been isolated and characterised, two different ssDNA viruses (CtenDNAV type-I and type-II), and two different ssRNA viruses (CtenRNAV type-I and type-II)^9,33^. However, the *C. tenuissimus* population in natural environments sustains its bloom size even in the presence of these viruses^10,11^. The tolerance to viral infection, along with their high growth rate, suggests that *C. tenuissimus* is to be a successful and ubiquitous species for maintaining primary productions in coastal environments. Knowledge on the relationship between *C. tenuissimus* and its viruses has gradually accumulated from the viewpoint of growth-physiology studies based on traditional culture experiments^17,34^, however, studies focusing on the aspect of cell biology are lacking. Here, we have explored the utility of genomic sequencing for this diatom species to further the current understanding regarding host-virus interactions at the molecular level. We believe that novel genomic discoveries can provide critical insights into the evolution of the diatom-virus relationship.

## Results

### Genome assembly and gene prediction

A total of 16.6 Gigabases (Gb) of sequence, providing a 150-fold coverage of the genome sequence, was obtained using the Illumina Miseq and Nanopore MinION platforms and assembled into 41 megabases (Mb) in a total of 85 scaffolds, ranging in size from approximately 1 kb to 4.46 Mb (Supplementary Table 1). In those scaffolds, telomeric sequence repeats (TTAGGG) were detected in 3 scaffolds (accession numbers BLLK01000038, BLLK01000056, and BLLK01000043) although the assembly is not yet at the chromosome level. The haploid genome size and its heterozygosity were estimated at 39.7 Mb and 1.56%, respectively (Supplementary Fig. 1) using the kmer-based statistic, and the size corresponding to the assembly. There is no huge insertion and inversion between haploid sequences (Supplementary Fig. 2). The accuracy of the genome assembly was confirmed by comparing it with the *T. pseudonana and T. oceanica* genomes using BUSCO software^35^*.* The 82 complete genes assigned by BUSCO were identified in the *C. tenuissimus* genome assembly, and the number was larger than that for the *Thalassiosira* species (Supplementary Table 2). Thus, a successful genome assembly of *C. tenuissimus* NIES-3715 was achieved. A summary of the genome structure is shown in Table 1. A total of 18,705 protein-coding genes were predicted in the haploid nuclear genome. Of the predicted proteins, 14,860 had significant similarities (e-value < 1e−5) to protein sequences in the non-redundant proteins database (nr), and 9,544 had recognisable InterPro domains. Complete chloroplast and mitochondrial genomes were identified from the assembly, with sizes of 116 kb and 36 kb containing 131 and 33 predicted genes, respectively (Table 1, Supplementary Fig. 3a,b). The chloroplast genome had synteny to a related species, *Chaetoceros simplex*^36^, with an identity of 99.4% (Supplementary Fig. 4a). In contrast, the mitochondrial genome in this study is the first reported in a *Chaetoceros* species (Supplementary Fig. 3b) and showed similarity to the mitochondrial genome of *T. pseudonana* (83.3% identity) which is classified with *Chaetoceros* as being Centrales (Supplementary Fig. 4b). All sequence reads obtained in this study were deposited in the DNA Data Bank of Japan (DDBJ) Sequence Read Archive under accession number DRA009158, and the assembly scaffolds for the nuclear genome, as well as the chloroplast and mitochondrial genomes of *C. tenuissimus* NIES-3715, were also deposited under accession numbers BLLK01000001-BLLK01000085, LC537471, and LC537470, respectively.

**Table 1:** Summary of genome assembly.

	*C. tenuissimus* NIES-3715	*T. pseudonana*	*P. tricornutum*
**Nuclear genome**			
Size (Mbp)	41.0	34.5	27.4
G+C content (overall %)	38.9	47	49
G+C content (coding %)	40.3	48	51
Protein-coding genes	18,705	11,242	10,402
Average gene size (bp)	1526	992	1527
**Chloroplast genome**			
Size (bp)	116,464	128,814	117,369
G+C content (overall %)	32.1	30.7	32.6
Protein-coding genes	131	144	130
tRNAs	75	33	30
**Mitochondrial genome**			
Size (bp)	36,047	43,827	77,356
G+C content (overall %)	30.8	30.1	35.0
Protein-coding genes	33	40	32
tRNAs	48	22	24

### EVLF in the nuclear genome of C. tenuissimus strains

**Figure 1:**
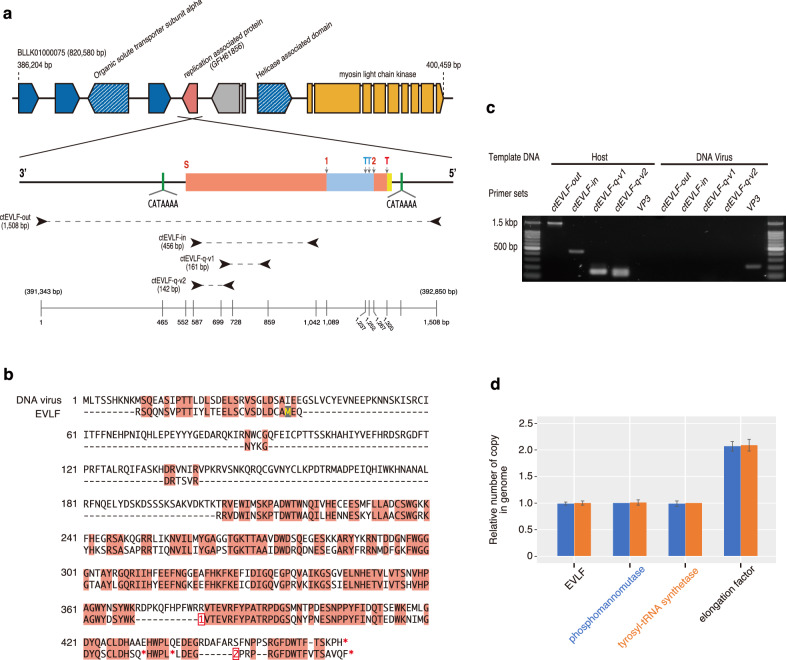
(**a**) Location and structure of the EVLF in the host genome. Gene directions are indicated by wide arrowheads at the end of each coding sequence. Blue and hatched blue indicate genes coding hypothetical proteins and hypothetical proteins possessing known domains, respectively. Orange indicates a myosin light chain kinase, and pink indicates a virus-like fragment. The structure of the EVLF sequence encoded on the reverse DNA strand is shown as a close-up. The pink and blue boxes indicate first and third open reading frames, respectively, due to single and double nucleotides were inserted in the positions marking on the boxes as “1” and “2”, and the “S” and “T” on the boxes refer to the start and terminal codons, respectively. A yellow box indicates a poly-(A) like sequence, and green boxes indicate a “CATAAAA” sequence. Black arrowheads indicate the amplification primer sets, ctEVLF-out-v1, ctEVLF-in-v1, ctEVLF-q-v1, and ctEVLF-q-v2, which amplified the 1508 bp, 456 bp, 161 bp, and 142 bp PCR products, respectively. (**b**) Alignment of amino acid sequences between the EVLF of *C. tenuissimus* NIES-3715 and a replication-associated protein in its infectious DNA virus. The alignment was constructed by MAFFT^63^. Pink boxes indicate consensus amino acid residues. Hyphens, asterisks, numbers in red boxes indicate gaps, termination codons, and nucleotide insertions, respectively. (**c**) PCR amplification for four primer sets using *C. tenuissimus* NIES-3715 and DNA virus. The amplification length of ctEVLF primer series is indicated (**a**) and that of VP3, which is coded in DNA virus genome, is 212 bp. No PCR bands using ctEVLF primer series were detected in DNA virus. (**d**) The relative number of gene copy in the *C. tenuissimus* NIES-3715 genome. The gene copies were relativized by phosphomannomutase (blue bar) or tyrosyl-RNA synthetase (orange bar).

An EVLF, encoding a predicted protein similar to the viral replication-associated protein in *C. tenuissimus* DNA virus, was found to be integrated between the predicted proteins in the nuclear genome (Fig. 1a). The 279 amino acids were encoded by 846 bases of the sequence and partially aligned with the sequence of the replication-associated protein in *C. tenuissimus* DNA virus SS12-43 V (64% identity; Fig. 1b). From this similarity, it was discovered that EVLF translation was frameshifted due to single- and two-base insertions and had two nonsense mutations in the sequence (Fig. 1a, b, and Supplementary Fig. 5). A poly-adenine (A) like sequence was observed downstream of the fragment and common sequence “CATAAAA” flanked the fragment (Fig. 1a).

**Figure 2:**
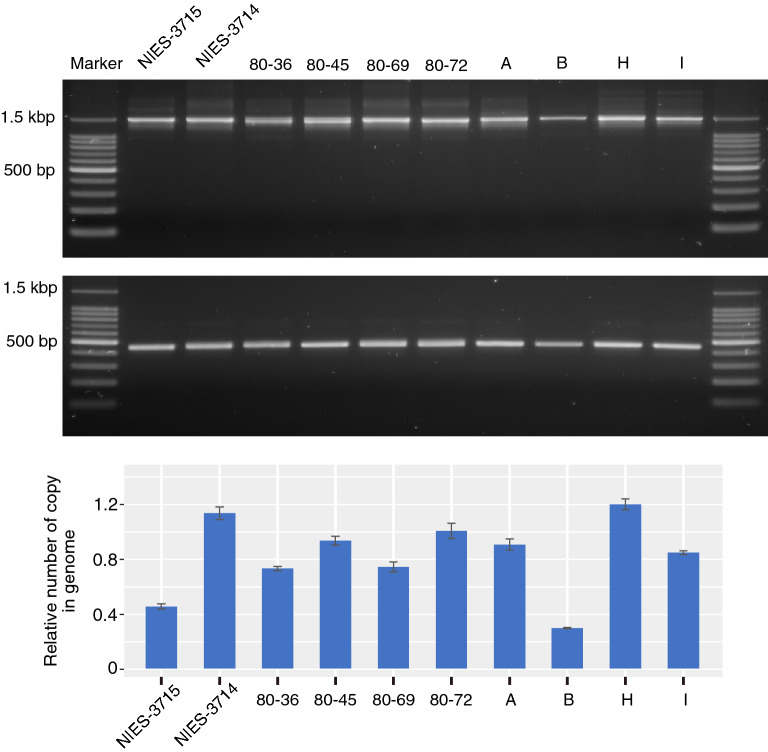
PCR amplification and copy number of the EVLF from the genomes of *C. tenuissimus* strains, isolated from the seawater sampling sites shown in Supplementary Fig. 7. The upper and lower PCR bands were amplified using ctEVLF-out-v1 and ctEVLF-in-v1 primer sets, respectively. The bar plot indicated relative numbers of EVLF copies in the genomes of each strain. The EVLF copies were relativised by the EVLF copy of NIES-3715 used ctEVLF-q-v1 primer and probe set. The EVLF copy numbers of all strains were measured using ctEVLF-q-v2 primer and probe set.

PCR analysis using ctEVLF-out-v1, ctEVLF-in-v1, ctEVLF-q-v1, and ctEVLF-q-v2 primer sets designed to amplify the EVLF over its 1.5, 0.5, 0.16, and 0.14 kbp length, respectively, revealed that specific bands were amplified in the EVLF region of *C. tenuissimus* NIES-3715 (Fig. 1c). Using DNA virus as a template for the primer sets of ctEVLF series, no amplification bands were detected (Fig. 1c). From the genome sequence information, EVLF was coded one copy in the haploid genome of *C. tenuissimus* NIES-3715. The quantity of the EVLF copy by the array-based digital PCR using ctEVLF-q-v1 primer set and the probe was estimated about 1 copy (Fig. 1d). This copy number was relativised against other single-copy genes, phosphomannomutase or tyrosyl-tRNA synthetase (Fig. 1d), estimated by BUSCO, and was half of the copy of elongation factor which was detected two copies in the haploid genome (Fig. 1d). For other strains, PCR analysis using ctEVLF-out-v1 and ctEVLF-in-v1 primer sets revealed that all strains of *C. tenuissimus* possessed the EVLF in their genomes (Fig. 2). To quantify the EVLF copies in the genomes of other strains, the ctEVLF-q-v2 primer and probe were designed in the consensus region of EVLF in all strains. However, this probe anneals only one side of the haploid region of *C. tenuissimus* NIES-3715 due to 10 bp of deletion exists in the annealing site of the other side of the haploid region. Therefore, EVLF copy in *C. tenuissimus* NIES-3715 was estimated at 0.5 copy (Fig. 2), and in other strains except for strain B were estimated about from 0.7 to 1.2 copies (Fig. 2). Although this probe anneals both haploids regions of all strains except for *C. tenuissimus* NIES-3715, strain B was only estimated at 0.3 copies (Fig. 2). These copy numbers were relativised by EVLF copy in NIES-3715 using ctEVLF-q-v1 primer and probe set.

### Phylogenetic distribution of EVLFs

**Figure 3:**
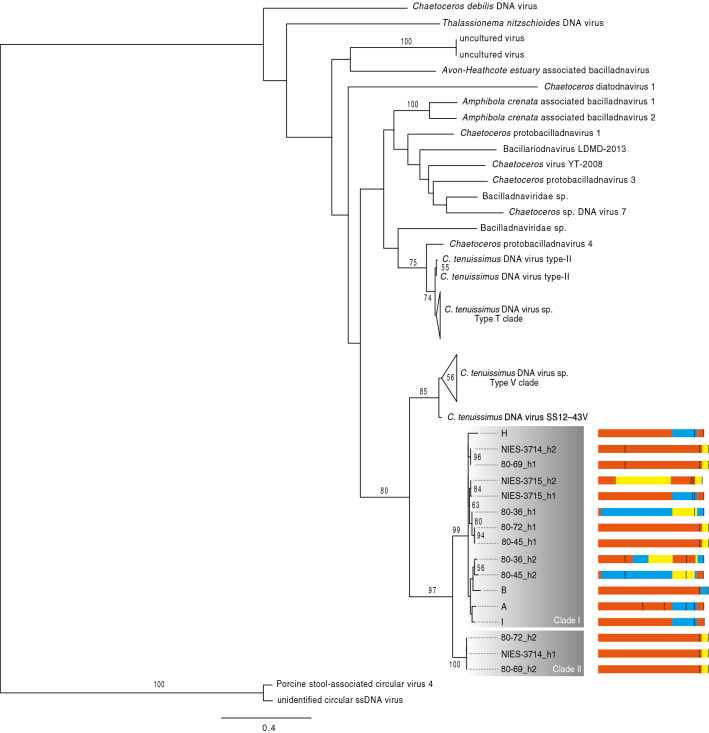
Maximum-likelihood trees of EVLFs with the replication-associated protein from bacilladnavirus. The EVLF fragment structures were shown in EVLF clade. The red, blue, and yellow boxes indicate the 1st, 2nd, and 3rd frames, respectively. The black vertical lines indicate the terminal codons. Bootstrap values greater than 50% are shown.

From the sequences of the cloned amplified gene fragments, a total of 16 clones with distinct sequences were obtained. These sequences were deposited in the DDBJ under accession number LC650336-LC650351. The translated amino acid sequences were aligned with the virus replication-associated protein, and the ML tree of these protein sequences indicated that all the EVLFs were clustered with ssDNA diatom virus replication-associated proteins (Fig. 3). In particular, the EVLFs were formed by the replication-associated proteins in the *C. tenuissimus* DNA virus type-V clade as a sister group, with moderate statistical support (BP = 80%; Fig. 3). In the EVLFs, the proteins were formed two clades with high statistical support (BP = 99% in clade I and BP = 100% in clade II; Fig. 3). Although all sequences had termination codons and/or frameshifts (Fig. 3), the evolutionary distances were close to each other within the clades (Fig. 3).

### Transcription of EVLF in C. tenuissimus NIES-3715

**Figure 4:**
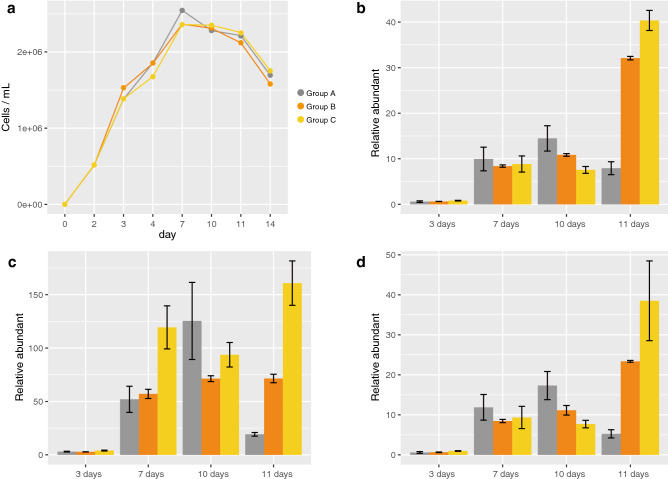
(**a**) Cell densities after 14 days of culture and the relative transcribe levels of the EVLF normalized by (**b**) *actin*, (**c**) *gapdh*, and (**d**) *elongation factor*. Cultures were performed in triplicate flasks (group a, b, c), and the line and bar colours in the plot correspond to the respective flasks.

For RT-qPCR analysis, the amplification efficiency of primer sets for EVLF and three reference genes, *actin*, *gapdh*, and *elongation factor*, were 93%, 94%, 95%, and 96%, respectively. Therefore, a relative abundance of transcription level for EVLF was calculated with ddCt method referring above three reference genes and comparing with 2 days cultures. In total 14 days of culture, the cell densities were rapidly increased until day 3, and then maintained >  × 10^6^ cells/ml to the end of the experiment (Fig. 4a). The maximum densities were ~ 2.6 × 10^6^ cells/ml at day 7. The cells for RT-qPCR analysis were sampled at 2- and 3-days cultures as an exponentially growing phase cell and at 7-, 10-, and 11-days cultures as a stationary phase cell. The EVLF was found to be transcribed in three replicate cultures of *C. tenuissimus* NIES-3715 at those sampling days (Fig. 4b–d). In particular, the relative transcription levels of the stationary phase were higher than those of the growth phase (*t-test* < 0.05, Fig. 4b–d). However, in the stationary phase of the cultures, the down-regulate of EVLF transcription was observed in group A from 10 to 11 days cultures (gray bars in Fig. 4b–d). As no amplification of the negative control was observed, all potentially contaminating DNA was completely digested in the samples (data not shown).

### Comparative genomics among different algae

**Figure 5:**
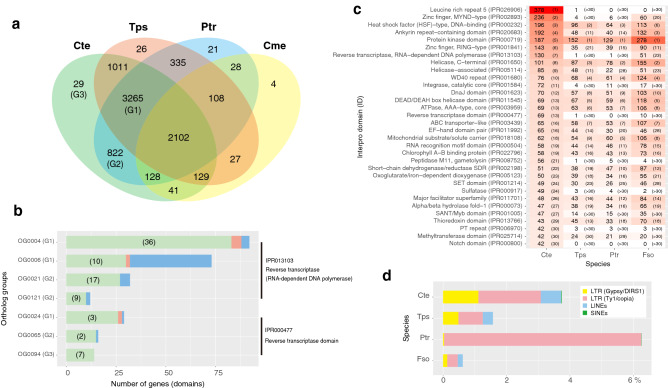
(**a**) Venn diagram of orthologous gene groups. Orthologous gene groups were identified from *C. tenuissimus* (Cte), *T. pseudonana* (Tps), *P. tricornutum* (Ptr), and *C. merolae* (Cme). The numbers in the Venn diagram indicate the number of orthologous gene groups. Reverse transcriptase domains (Interpro ID, IPR013103 and IPR000477) were included in the ortholog gene groups (G1, OG0004, OG0006, and OG0024; G2, OG0021, OG0121, and OG0065; G3, OG0094). (**b**) The number of genes in the above ortholog gene groups. The parentheses indicate the number of genes that possessed reverse transcriptase domains (IPR013103 and IPR000477). (**c**) Top 30 of the Interpro domains detected in the *C. tenuissimus* genome. The gene numbers are in descending order based on Cte and the numbers in parentheses indicate their ranking. The shade of red becomes lighter as the gene number decreases. Fso, *Fistulifera solaris. *(**d**) The percentage of retroelements in the four species genomes predicted by RepeatMasker.

The orthologous genes among *Chaetoceros tenuissimus* NIES-3715, *Thalassiosira pseudonana*, *Phaeodactylum tricornutum*, and *Cyanidioschyzon merolae* were identified from a total of 18,705, 11,673, 10,408, and 4803 protein sequences, respectively. The genes included in the 2102 ortholog groups were common among these organisms (Fig. 5a). In the *C. tenuissimus* genes, 3265 ortholog groups were common in both *T. pseudonana* and *P. tricornutum*, while in the other groups a total of 1011 and 822 were common in *T. pseudonana* and *P. tricornutum*, respectively (Fig. 5a). The specific paralog genes in *C. tenuissimus* also formed 29 groups (Fig. 5a).

In the ortholog group, OG0000, 385 paralog genes in *C. tenuissimus* were most abundant among these organisms and its 376 amino acid sequences possessed a leucine rich repeat 5 domain (IPR026906; Supplementary Table 3). Subsequently, seven ortholog groups, OG0004, OG0006, OG0021, OG0024, OG0065, OG0094, and OG0121, noticeably formed groups with genes possessing reverse transcriptase domains (IPR013103 and IPR000477), although the number of genes that contained these domains were not equal to that of the paralog genes (Fig. 5b). In the above ortholog groups, except for OG0006, the genes in *C. tenuissimus* were more abundant than those of other organisms (Fig. 5b).

The number of genes that possessed a functional domain is shown in Fig. 5c. The genes that possessed a protein kinase domain (IPR000719) were found to be abundant in all organisms, while genes that possessed the following four distinct domains, leucine rich repeat 5 (IPR026906), zinc finger, MYND-type (IPR002893), heat shock factor (HSF)-type, DNA-binding (IPR000232), and ankyrin repeat-containing domain (IPR020683; Fig. 5c), were more abundant in *C. tenuissimus* than in those of the other three diatom species. Moreover, the number of genes that possessed the following eight domains in *C. tenuissimus* was notably higher than that in other organisms; leucine rich repeat 5 (IPR026906), zinc finger, RING-type (IPR001841), reverse transcriptase, RNA-dependent DNA polymerase (IPR013103), integrase, catalytic core (IPR001584), reverse transcriptase domain (IPR000477), peptidase M11, gametolysin (IPR008752), sulfatase (IPR000917), and notch domain (IPR000800; Fig. 5c).

### Transposable elements

Although the total number of retroelements in *C. tenuissimus* was less than that in *P. tricornutum*, three retroelements of *C. tenuissimus* were detected at higher levels than those in other organisms: short interspersed nuclear elements (SINEs, 0.02%), long interspersed nuclear elements (LINEs, 0.64%), and Gypsy/DIRS1 of long terminal repeat elements (1.11%; Fig. 5d).

## Discussion

Diatoms and their infectious viruses seem to be able to coexist in natural waters and are closely related to each other^10,11^. To understand the ecological relationships between host diatoms and their viruses, we sequenced the *C. tenuissimus* genome. Surprisingly, a predicted gene having a similarity to a putative replication-associated protein of its infectious ssDNA virus was found to be integrated between two coding sites (Fig. 1a). The predicted EVLF protein sequence was highly similar to the mid-region of the replication-associated protein in *C. tenuissimus* ssDNA virus SS12-43V, which infects and lyses *C. tenuissimus* NIES-3715. Moreover, a frameshift caused by single- and two-base insertions, as well as nonsense mutations, was observed in the sequence (Fig. 1b). Therefore, the integration of the virus replication-associated gene appears to have incomplete and is seemed to be not functional. Like *C. tenuissimus*, an integrated virus fragment has been shown in the diatom *P. tricornutum* genome and its transcription has been detected by an EST analysis^37^. However, this fragment is not similar to the replication-associated protein in *C. tenuissimus* but instead is similar to a viral replication gene identified only from viral metagenomic data (data not shown). Therefore, the integration of an extant closely related infectious virus fragment into its host genome is rare in diatoms.

When a virus first infects the host, its replication relies on the host cellular machinery^38^. In particular, the genes of DNA viruses must be transcribed as mRNA^38^. The ssDNA diatom viruses encode three putative proteins in their genomes^33^. During the infection, there are several key molecular processes required for the successful integration of a viral gene into the host genome. A Bornavirus-like nucleoprotein gene is integrated into mammalian genomes, and this integration is thought to be mediated through mRNAs by retrotransposons, which are mobile genetic elements with their reverse transcriptase (RT), such as LINEs^39^. The banana genome also contains an integrated infectious virus gene, and it is thought to have been integrated by the Ty3/gypsy retrotransposon, which is a retroelement with long terminal repeats^40^. In the *C. tenuissimus* genome, the number of proteins that possessed an RT domain was higher than that in *T. pseudonana*, *P. tricornutum*, and *Fistulifera solaris* (Fig. 5c). Similarly, the percentage of predicted retroelements in *C. tenuissimus* was higher than that in *T. pseudonana* and *F. solaris* (Fig. 5d). Notably, the percentage of LINEs in the *C. tenuissimus* genome was the highest among the different species (Fig. 5d). In addition, considering that the number of paralog genes possessing or lacking an RT domain in *C. tenuissimus* was higher than that in other species, it is assumed that the *C. tenuissimus* genome contains more retrotransposons (Fig. 5b). These results indicated that more retrotransposons have been copied and integrated into the genome, which might be a factor in explaining the large genome size of *C. tenuissimus*. It is, therefore, possible that the retrotransposons possessing the RT domain converted the mRNA of the virus replication-associated gene into cDNA during the infection process. However, this then leads to the question of how the cDNA of the virus replication-associated gene integrates into *C. tenuissimus* genome. In general, once retroelements enter the nucleus, an endonuclease encoded by a LINE makes a single-stranded endonucleolytic nick in genomic DNA at a degenerate consensus sequence (5′-TTTT/A-3′, with “/” indicating the scissile phosphate), exposing a poly-(T) tail and a 3′ hydroxyl group that serves as a primer for the reverse transcription of retroelement mRNA, and subsequent integration^41,42^. The integrated retroelements have a structural hallmark in that they are flanked by variable size target site duplications (TSDs) ^41,42^. This structural hallmark was observed in the region of the integration of EVLF in the *C. tenuissimus* strains (Supplementary Fig. 6a,b). A poly-(A) like sequence was observed downstream of the stop codons, which corresponds to the terminus of the virus replication-associated gene (Supplementary Fig. 6b). In addition, the endonuclease cleavage sites “5′-TTTTATG-3′” flanked the EVLF and were characterised as TSDs (Fig. 1a and Supplementary Fig. 6). In addition, a 5′ truncation and point mutations within the integrated retrotransposon are characteristic of integration by LINEs^42^. These structural hallmarks were also observed in the EVLFs of the *C. tenuissimus* strains but not in the *P. tricornutum* genome^37^. In the case of *C. tenuissimus*, this evidence suggested that the virus replication-associated gene was possibly integrated into the *C. tenuissimus* genome by host LINEs.

In general, endogenous viral elements interfere with any step of viral infection, acting as restriction factors^43^. In the culture experiments without a virus inoculation, it was clear that transcription of EVLF in the stationary phase was higher than that in the growth phase. However, down-regulate of the transcription in a part of cell culture was detected (Fig. 4b–d). Because cell concentration in group A was decreased from 7- to 10-days, it was considered that extinct cells may be abundant in the cell culture and made to decrease the transcription. CtenDNAV type-II was inoculated into the host cells at the growth phase, and the cell density decreased after the cells reached the stationary phase^33^. In short, the inoculated cells maintained a high growth rate, suggesting that *C. tenuissimus* can resist virus infection^44^. However, considering this phenomenon together with the fact that EVLF is transcribed, it seemed that there was no correlation between the expression of EVLF and resistance against the virus. To date, RNA silencing has been shown to act as a defense response against viral infections in plants^45–47^ and mosquitos^48^, Moreover, this gene silencing mechanism functions in the diatom, *P. tricornutum*^49^. The *C. tenuissimus* genome also encoded genes important in RNAi function, such as genes encoding Dicer and Argonaute proteins (accession nos. GFH46084.1 and GFH61989.1, respectively). Although it remains whether EVLF functions to RNA silencing against viral proliferation, the potential function of EVLF for the virus should be clarified in the future.

ssDNA viruses infecting *C. tenuissimus* are classified into two types (type-T and type-V) based on the sequences of replication-associated proteins^50^. The EVLFs were certainly derived from the type-V clade of *C. tenuissimus* DNA virus (Fig. 3). Although the geographic distribution of these strains is different in Japanese coastal waters (Supplementary Fig. 7), the EVLF was highly conserved among strains, moreover, all strains were found to have EVLF at the same locus (Fig. 2). These results indicate that the ancestor of *C. tenuissimus* had acquired the replication-associated gene from one type of virus. The number of EVLF copies in *C. tenuissimus* NIES-3715 was confirmed by comparing with other single-copy and two copies genes. As a result, one copy genes including EVLF were estimated at 1 copy (Fig. 1d). This copy number was doubled using another primer and probe set, ctEVLF-q-v2 (0.5 copy; Fig. 2). Therefore, we considered that one copy of EVLF was integrated into the genome. EVLF copies of other strains except strain B were also about from 0.7 to 1.2 copies (Fig. 2). In this case, because the copy number was not relativised by the number of housekeeping gene copies in each strain but the number of EVLF copies in NIES-3715, a range of copy numbers might have varied. Like NIES-3715, we considered that the other strains were single-copy strains except for strain B. EVLF copy in strain B was estimated at 0.3 copies (Fig. 2). It was similar to the copy number of NIES-3715 using ctEVLF-q-v2 primer and probe set. In addition, the intensity of the PCR band of EVLF in strain B was lower than those in other strains (Fig. 2), and only one type of EVLF cloned sequence was detected (Fig. 3). Therefore, strain B might have an EVLF on one side of the haploid genome. The EVLF integration within the strains was varied. This result shows the fact that the EVLFs remain encoded in their genome as a fossil. Although the enigma of the survival strategy against the infectious viruses used by *C. tenuissimus* remains to be solved, the populations have survived to date. We consider that the EVLF integration might have provided an advantageous condition for the host in the evolutionary relationship between diatom and virus, and remains to be interested whether the sequence variations of EVLF influence viral infectivity.

In this study, we sequenced the *C. tenuissimus* genome and discovered that a replication-associated gene associated with its infectious virus has been integrated into the genome. This discovery represents the few cases in diatoms and indicated that the virus fragment might have integrated into other diatom genomes, that is, a close evolutionary relatedness between diatom and virus will start to shed light.

## Methods

### DNA extraction and DNA library construction

The *C. tenuissimus* strain NIES-3715 was isolated from the Seto Inland Sea, Japan (Supplementary Fig. 7) in Aug 2002. This strain has been subcultured to date and used for genome sequence. To check for bacterial contamination, the cultures were observed using epifluorescence microscopy after staining with SYBR-Gold. Briefly, the lysate was fixed with glutaraldehyde at a final concentration of 1%, and SYBR-Gold (Thermo Fisher Scientific, Waltham, MA, USA) was added to each fixed sample at a final dilution of 1.0 × 10^–4^ of the commercial stock. The stained samples were filtered onto 0.2-µm polycarbonate membrane filters (Nuclepore membrane; Cytiva, Sheffield, UK), after which the filters were mounted on a glass slide with a drop of low-fluorescence immersion oil, and covered with another drop of immersion oil and a coverslip. The slides were viewed at 1000 × magnification with an Olympus BX50 epifluorescence microscope. The axenic algal cultures were grown in a modified SWM3 medium enriched with 2 nM Na_2_SeO_3_^51^ under a 12/12-h light–dark cycle at 20 °C. Light irradiance was 850 µmol m^−2^ s^−1^ using white LED illumination. The algal strain was cultured for 7 days. Approximately 3 × 10^6^–5 × 10^6^ cells/ml in the stationary phase were used for DNA extraction. The cells in the cultures were harvested by centrifugation at 860×*g* and 4 °C for 15 min, after which the cell pellets were stored at – 80 °C until analysis. DNA was extracted from the samples using a DNeasy Plant Mini Kit (Qiagen, Valencia, CA, USA), according to the manufacturer’s instructions. DNA libraries for paired-end and mate-paired sequencing were constructed in accordance with KAPA Hyper Prep Kit (F. Hoffmann-La Roche Ltd., Basel, Switzerland) and Nextera Mate Pair Sample Prep Kit (Illumina, Inc. San Diego, CA, USA), respectively. These libraries were sequenced into 300 bp paired-end reads using MiSeq (Illumina, Inc.) at the Japan Agency for Marine-Earth Science and Technology, Yokosuka, Japan.

For long-read sequencing of genomic DNA using MinION (Oxford Nanopore Technologies, Oxford, UK), total nucleic acid was extracted from the pellet using the DNAs-ici!-F (RIZO Inc., Tsukuba, Japan), according to the manufacturer’s protocol. To extract genomic DNA from the total nucleic acid sample, the sample was treated with RNase A (Nippon Gene, Tokyo, Japan) and subsequently purified with phenol/chloroform before the construction of a DNA sequencing library. The sequencing library was constructed using the ligation sequencing kit (SQK-LSK109, Oxford Nanopore Technologies) and sequenced by MinION, according to the respective manufacturer’s instructions. After sequencing, base-calling was performed with Albacore (v2.3.1, Oxford Nanopore Technologies).

### De novo* genome assembly*

To estimate the genome size and heterozygosity, k-mer counting was performed using the short paired-end reads and the Jellyfish programme^52^. The histogram of 21mer counts was visualised using GenomeScope^53^.

Hybrid assembly of all Illumina short reads and MinION reads was performed using MaSuRCA^54^ (v3.3.0) with default parameters. The haploid genome sequence was constructed from the assembled genome using HaploMerger2^55^ (v20180603) with default parameters. The assembly quality was evaluated by QUAST^56^. To evaluate the assembly accuracy, single-copy ortholog genes were searched using BUSCO^35^ with alveolata_stramenophiles_ensembl datasets.

Two haploid pairs were aligned using MUMmer^57^ with default parameters. To visualise easily, the alignment data were filtered using the delta-filter command in MUMmer with “-q” and “-r” options and under default settings for all other options. The dot plot was drawn using mummerplot with default parameters.

### Gene prediction and annotation

To predict the gene regions in the genome sequence, we first obtained the complete open reading frames (ORFs) from RNAseq (Supplementary Note, DDBJ Sequence Read Archive under accession number DRA011082). In RNAseq, the de novo assembly procedure was performed following that described by Hongo et al.^58^. The complete ORFs were extracted from the assembled sequences and translated using TransDecoder^59^ with default parameters. Next, the quality-controlled paired-end reads of RNAseq were mapped to the assembled genome using TopHat2^60^ with default parameters. Using the mapping data and the protein sequences of complete ORFs, the gene model was predicted using BRAKER2^61^ (v2.1.0) with default parameters. Proteins predicted from the gene model were annotated based on their homology to sequences in the nr database from NCBI using the BLASTP program with a threshold e-value of < 1e−5, and protein domains were found using Interproscan with a threshold e-value of < 1e−5. The complete chloroplast and mitochondrial genomes were automatically annotated by GeSeq annotation server^62^.

### Quantification of gene copy in the nuclear genomes using array-based digital PCR

PCR amplification was conducted in a reaction mixture with 34.8 µl final volume for two array chips, containing 1 ng of the extracted DNA, 1 × QuantStudio 3D Digital PCR Master mix (Thermo Fisher Scientific), 0.86 µM of each primer, and 0.29 µM of TaqMan probe. Each sample was analysed by 4 chips. PCR and data acquisition were conducted in accordance with the manufacturer’s instructions. Data analysis was performed using the QuantStudio 3D Analysis Suite Software. The sequences of both TaqMan probes and specific primers are shown in Table 2.

**Table 2:** Primer and probe sequences used in this study.

Name	Direction	Sequence (5′ → 3′)	Length (bp)
**Primers**			
ctEVLF-out-v1	Forward	GCAAACACGTKTGTTGATATATCGG	1508
	Reverse	CGATCCTCTTGAAGACCCAGT	
ctEVLF-in-v1	Forward	AAGAAGAAGAGTCGACTGGATCAAC	456
	Reverse	ACAATAACGGTCTCATGATTGAGC	
ctEVLF-q-v1	Forward	AAGTATCACAAGTCACGCTCTGC	161
	Reverse	TCCATATTACGTCTGAAGTAGCGAG	
ctEVLF-q-v2	Forward	AAGAAGAAGAGTCGACTGGATCA	142
	Reverse	AGCACTTGCAGAGCGTGACT	
Phosphomannomutase	Forward	GTGGAACCTTTATTGAATTCCGTAG	188
	Reverse	AATCTGTCCTCCAATGGAATATGTC	
Tyrosyl-tRNA synthetase	Forward	GTAAGAGTGATCCTGACAGTGCTG	266
	Reverse	GATTGGTTCAAATTCGGAGTAGG	
Actin	Forward	CTGGATGTGTTCTTGATTCTGGAG	168
	Reverse	CTGCAGTAGTGGTGAGGGAGTATC	
Gapdh	Forward	CATTTTCCTCCAGCTCCTTCTC	261
	Reverse	GGAAGTCTCCATCAATATCTACGGTTC	
Elongation factor	Forward	GAAACTCCGTTGGTATGTCCATC	163
	Reverse	CAGTATCCGGTCTTGAGTACTCCAG	
**Probes**			
ctEVLF-q-v1		FAM-CTGCAGCCA-ZEN-TAGACTGGGATAGGCAAGAC-lowa Black® FQ	
ctEVLF-q-v2		FAM-ATGAATCTA-ZEN-AATATTTGTTAGCTGCTTGCTCCTGG-lowa Black® FQ	
Phosphomannomutase		FAM-AAGAAATTG-ZEN-CTCTCGTGAGGAACGTAATGACTATG-lowa Black® FQ	
Tyrosyl-tRNA synthetase		FAM-TAAGGAAGA-ZEN-AACTGATGCTGGAAAGGAGTCTATGC-lowa Black® FQ	
Actin		Yakima Yellow®-ATCTATGAG-ZEN-GGATATGCTCTTCCACACGCTGTAGT-lowa Black® FQ	
Gapdh		HEX-CACTGGAAT-ZEN-GGGAGTTAACGGATTCGGAC-lowa Black® FQ	
Elongation factor		FAM-CATCTACAA-ZEN-GGAGTCTGAGGGAGACTGTCTTCCAG-lowa Black® FQ	

Since the results were expressed by copies per microliter, the number of gene copy was relativised based on the result of the number of single-gene copies.

### Confirmation of an EVLF in the nuclear genome

To analyse the EVLF in the *C. tenuissimus* genome, we used nine other strains of this diatom species other than strain NIES-3715 (Supplementary Fig. 7). One milliliter of a stationary growth phase *C. tenuissimus* culture was centrifuged at 17,400×*g* for 3 min at 4 °C. The resulting diatom cell pellets were then preserved at − 80 °C until analysis. DNA was extracted from stored cell pellets using the DNeasy Plant Mini Kit (Qiagen), according to the manufacturer’s instructions. The EVLF was amplified using each primer pair, ctEVLF-out-v1 and ctEVLF-in-v1 (Fig. 1a and Table 2).

PCR amplification was conducted in a reaction mixture with a 25 µl final volume, containing 1 µg DNA, 1 × *ExTaq* buffer (Takara, Shiga, Japan), 200 nM dNTPs, 0.2 µM of each primer, and 0.025 U *ExTaq* DNA polymerase. PCR was conducted using GeneAmp PCR system 9700 with the following cycle parameters: initial denaturation phase of 98 °C for 2 min, followed by 30 cycles of 98 °C for 10 s, 60 °C for 15 s, and 72 °C for 90 s. The PCR products were then electrophoresed on 1% (w/v) agarose ME gels (Wako Pure Chemical Industries, Osaka, Japan), and the nucleic acids were visualised using SYBR Safe DNA gel stain (Thermo Fisher Scientific). PCR amplicons of approximately 1.5 kb were excised, and their nucleic acids were extracted (NucleoSpin® Gel and PCR Clean-up; Macherey–Nagel GmbH and Co., KG, Düren, Germany). The PCR products were ligated into the pGEM-T Easy vector (Promega, Madison, WI, USA) and transformed into *Escherichia coli* DH5α-competent cells (Toyobo, Japan). Sequencing was conducted using the dideoxy method with ABI PRISM 3130 Genetic Analyzer (Thermo Fisher Scientific).

### Phylogenetic analysis of EVLFs and virus replication-associated genes

The genome sequence of EVLF was obtained for ten strains of *C. tenuissimus* (Supplementary Fig. 7) using the above genome sequence confirmation. To clarify the evolutionary relationship of the EVLFs, a maximum-likelihood (ML) tree analysis was conducted. The virus protein sequences in the NCBI database were retrieved based on similarity to the EVLF using the BLASTP program. These accession numbers are shown in Supplementary Table 4. All retrieved protein sequences and the EVLF were aligned using MAFFT^63^(v7.212) with default parameters, and all stop codons and the frame-shifted amino acids in the alignment were removed by manual editing. Gaps were automatically trimmed using trimAl^64^ using the ‘-automated1’ command option and default settings for all the other options. The best-fit evolutionary model for the optimum alignment was calculated using ModelFinder^65^ and the Akaike information criterion. The ML tree was inferred from an evolutionary model using RAxML^66^ (v8.2.4) with 100 bootstrap replicates.

### RT-qPCR analysis

The axenic algal cultures of *C. tenuissimus* strain NIES-3715 were grown in a modified SWM3 medium under a 12/12 h light–dark cycle of ca. 600 to 700 µM of photons m^−2^ s^−1^, using cool white, fluorescent illumination at 25 °C for 3 days. For the RT-qPCR analysis, preconditioned cultures were inoculated into 1-l of fresh SWM3 medium at a final density of 978 cells/ml using a 2-l polycarbonate Erlenmeyer flask (431255; Corning Inc, Glendale, AZ, USA). This experiment was performed in triplicate. The cultures were subsampled at the logarithmic growth phase (day 2, 3, 4, and 7) and at stationary phase (day 10, 11, and 14). On each sampling day, diatom cells in the cultures were retained on 0.4-µm polycarbonate membrane filters (Nuclepore membrane; Cytiva), and then the cells were immersed in RNAlater (Thermo Fisher Scientific) for 5 min. After the incubation, RNAlater was filtered out. The number of diatom cells on the filters ranged from 10^7^ to 10^8^ cells per filter, which were frozen in liquid nitrogen and stored at – 80 °C until analysis.

The retained filters containing the diatom cell samples were cut into small pieces in the TRIzol reagent (Thermo Fisher Scientific), and total RNA was extracted using a TRIzol Plus RNA Purification Kit (Thermo Fisher Scientific), with PureLink DNase (Thermo Fisher Scientific) digesting any contaminating DNA, in accordance with the manufacturer’s instructions. Moreover, to completely digest any contaminating DNA, DNase treatment was performed using a TURBO DNase free kit (Thermo Fisher Scientific). The quantity of the total RNA was measured using a Qubit RNA HS assay kit (Thermo Fisher Scientific). cDNA was constructed from 1 µg of total RNA using an oligo(dT)_15_ primer and SuperScript IV Reverse transcriptase (Thermo Fisher Scientific), in accordance with the manufacturer’s instructions.

PCR amplification was conducted in a reaction mixture with a 20 µl final volume, containing 1 µl cDNA, 1 × Probe qPCR mix (Takara), 0.2 µM of each primer, and 0.2 µM of TaqMan probe. qPCR was conducted using QuantStudio 3 (Thermo Fisher Scientific) with the following cycle parameters: initial denaturation phase of 95 °C for 30 s, followed by 45 cycles of 95 °C for 5 s, 60 °C for 30 s. All samples and a no-template control were analysed in triplicate. RNA template as a negative control was conducted with the above same condition. The sequences of both TaqMan probes and specific primers are shown in Table 2.

### Identification of orthologous genes

Protein sequences from *T. pseudonana*, *P. tricornutum*, and *Cyanidioschyzon merolae* were retrieved from public databases (accession no. GCF_000149405.2, GCF_000150955.2, and GCF_000091205.1, respectively). Orthologous gene groups in all the protein sequences, including those in *C. tenuissimus*, were found using OrthoFinder^67^ with default parameters. Protein domains in the sequences of reference organisms (*T. pseudonana*, *P. tricornutum*, and *F. solaris*, accession no. GCA_002217885.1) were found using InterProscan using a threshold e-value of < 1e−5.

### Prediction of transposable elements

Transposable elements (TEs) in the *C. tenuissimus* genome were predicted using RepeatModeler2^68^ and RepeatMasker^69^ programmes with default parameters. To compare the TEs statistically among diatoms, the genomes of *T. pseudonana*, *P. tricornutum*, and *F. solaris* were analysed using the same programmes and parameters and compared to TEs that have already been reported for these three genomes^70,71^.

## Supplementary Information

Supplementary Information.

Supplementary Figures.

Supplementary Tables.

## Data Availability

Sequence data generated during the current study are available in DDBJ repository, under accession number DRA009158, and the assembly data analysed during the current study are also available in the DDBJ repository, under accession numbers BLLK01000001-BLLK01000085 (nuclear genome), LC537471 (chloroplast genome), and LC537470 (mitochondrial genome). Sixteen distinct sequences of EVLF are under accession number LC650336-LC650351.
