# Supplementary material for: The genome of the diatom Chaetoceros tenuissimus carries an ancient integrated fragment of an extant virus
Source: Sci Rep. 2021 Nov 24;11:22877. doi: 10.1038/s41598-021-00565-3 (PMC8613185; doi:10.1038/s41598-021-00565-3)
Supplement: Supplementary file 1 — Supplementary Information. [file 41598_2021_565_MOESM1_ESM.docx]

**Supplementary Note**

**Article title: The genome of the diatom *Chaetoceros tenuissimus* carries an ancient integrated fragment of an extant virus**

Yuki Hongo^1^*, Kei Kimura^2^, Yoshihiro Takaki^3^, Yukari Yoshida^3^, Shuichiro Baba^4^, Genta Kobayashi^2^, Keizo Nagasaki^5^, Takeshi Hano^6^ and Yuji Tomaru^6^*

^1^Fisheries Resources Institute, Japan Fisheries Research and Education Agency, 2-12-4 Fukuura, Kanazawa, Yokohama, Kanagawa 236-8648, Japan

^2^Faculty of agriculture, Saga University, 1 Honjo-machi, Saga 840-8502, Japan

^3^Super-cutting-edge Grand and Advanced Research Program, Japan Agency for Marine-Earth Science and Technology, 2-15 Natsushima-cho, Yokosuka, Kanagawa 237-0061, Japan

^4^United Graduate School of Agricultural Sciences, Kagoshima University, 1-21-24 Korimoto, Kagoshima 890-0065, Japan

^5^Faculty of Science and Technology, Kochi University, 200 Otsu, Monobe, Nankoku, Kochi 783-8502, Japan

^6^Fisheries Technology Institute, Japan Fisheries Research and Education Agency, 2-17-5 Maruishi, Hatsukaichi, Hiroshima 739-0452, Japan

***Corresponding authors**

Yuki Hongo

Fisheries Resources Institute, Japan Fisheries Research and Education Agency

2-12-4 Fukuura, Kanazawa, Yokohama, Kanagawa 236-8648, Japan

Tel: +81-45-788-7673

Fax: +81-45-788-5001

Email: [hongoy@affrc.go.jp](mailto:hongoy@affrc.go.jp)

ORCID: <https://orcid.org/0000-0003-1434-1220>

Yuji Tomaru

Fisheries Technology Institute, Japan Fisheries Research and Education Agency

2-17-5 Maruishi, Hatsukaichi, Hiroshima 739-0452, Japan

Tel: +81-829-55-3529

Fax: +81-829-54-1216

Email: tomaruy@affrc.go.jp

**Data Description**

Total RNAs of *Chaetoceros tenuissimus* NIES-3715 were extracted from 4 replications under complete and phosphate deficiency mediums at the logarithmic and stationary growth phases. Average 11.5 million raw paired-end sequence data were yielded from the 16 cDNA libraries using the Illumina HiSeq 2500 platform and assembled into a *de novo* transcripts using Trinity^1^. All raw sequence data were deposited at DNA Data Bank of Japan (DDBJ) Sequence Read Archive under accession no. DRA011082.

**RNA extraction and cDNA library construction.**

Algal cultures were grown in modified SWM3 medium enriched with 2 nM Na_2_SeO_3_ ^2^ under a 12/12-h light-dark cycle at 20°C. Light irradiance was 850 µmol m^-2^ s^-1^ using white LED illumination. The final concentration of additive phosphate (NaH_2_PO_4_ H_2_O) for SWM3 is 100 μM (phosphate-replete SWM3 media). In this study, we also prepared a phosphate deficiency SWM3 medium with a final phosphate concentration of 3.3 μM (phosphate-deficient SWM3 media).

Exponentially growing cultures of *C. tenuissimus* NIES-3715 (1.1×10^6^ cells/mL) were respectively inoculated into eight 250 mL phosphate-replete and -deficient SWM3 medium (total 16 flasks) (0.36% v/v) at a final cell concentration of 4.0×10^3^ cells/mL. Cell counts were carried out with an image-based cytometric analysis with the Tali image-based cytometer (Thermo Fisher Scientific Ltd., Waltham, MA, USA) using the red channel (excitation filter, 543/22 nm; longpass emission filter, 585 nm). The cell size range in a bright field, red fluorescent threshold, circularity, and sensitivity were set at 3 µm – 20 µm, 1200, 8, and 9, respectively. A 25-μL aliquot of a *C. tenuissimus* culture was placed in the disposable counting slides (Thermo Fisher Scientific Ltd., Waltham, MA, USA) and cells were counted according to supplier instructions after standing for 10 min in the dark at room temperature^3^. Four out of the eight flasks after 2 days of incubation for phosphate-replete and -deplete conditions were used for mRNA analysis of exponential growth phase conditions of *C. tenuissimus*, and the other four flasks were sampled at 7 days for stationary phase condition.

The cells in 30 mL of the sample were retained onto 0.4 μm polycarbonate membrane filters (GE Healthcare). They were put into 1.5 mL tubes and rapidly frozen with liquid nitrogen. The samples were stored at −80°C until analysis. RNA from the cell samples was extracted with RNeasyPlus Mini Kit (Qiagen, Valencia, CA) according to the manufacture’s instruction. Total RNA concentration was measured with Nanodrop 2000 (Thermo Fisher Scientific). The concentration and quality of mRNA samples were examined using an Agilent 2100 Bioanalyzer. TruSeq Stranded mRNA Sample Prep Kit (Illumina) was used for cDNA library construction according to the manufacture’s instruction, and the libraries were sequenced by 100 bp paired-end reads using the Illumina HiSeq 2500 platform.

Reference

1. Haas, B. J. *et al.* De novo transcript sequence reconstruction from RNA-seq using the Trinity platform for reference generation and analysis. *Nat. Protoc.* **8**, 1494–1512 (2013).

2. Imai, I., Itakura, S., Matsuyama, Y. & Yamaguchi, M. Selenium Requirement for Growth of a Novel Red Tide Flagellate *Chattonella verruculosa* (Raphidophyceae) in Culture. *Fish. Sci.* **62**, 834–835 (1996).

3. Tomaru, Y. & Kimura, K. Rapid quantification of viable cells of the planktonic diatom *Chaetoceros tenuissimus* and associated RNA viruses in culture. *Plankt. Benthos Res.* **11**, 9–16 (2016).
