# Supplementary material for: The genome of the diatom Chaetoceros tenuissimus carries an ancient integrated fragment of an extant virus
Source: Sci Rep. 2021 Nov 24;11:22877. doi: 10.1038/s41598-021-00565-3 (PMC8613185; doi:10.1038/s41598-021-00565-3)
Supplement: Supplementary file 2 — Supplementary Figures. [file 41598_2021_565_MOESM2_ESM.pdf]

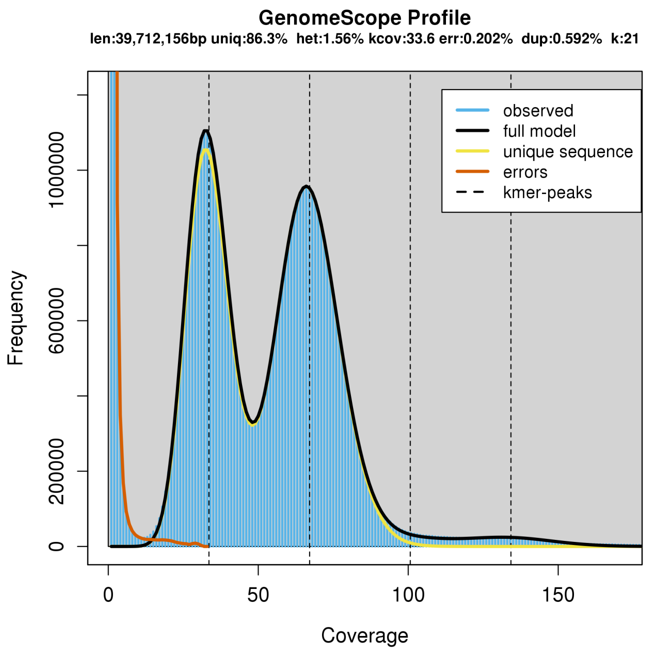

Supplementary Figure 1. Histogram of the kmer coverage in the genome sequences. The two peaks in coverage indicated that *C. tenuissimus* NIES-3715 is a diploid (Reference No.53 in the manuscript).

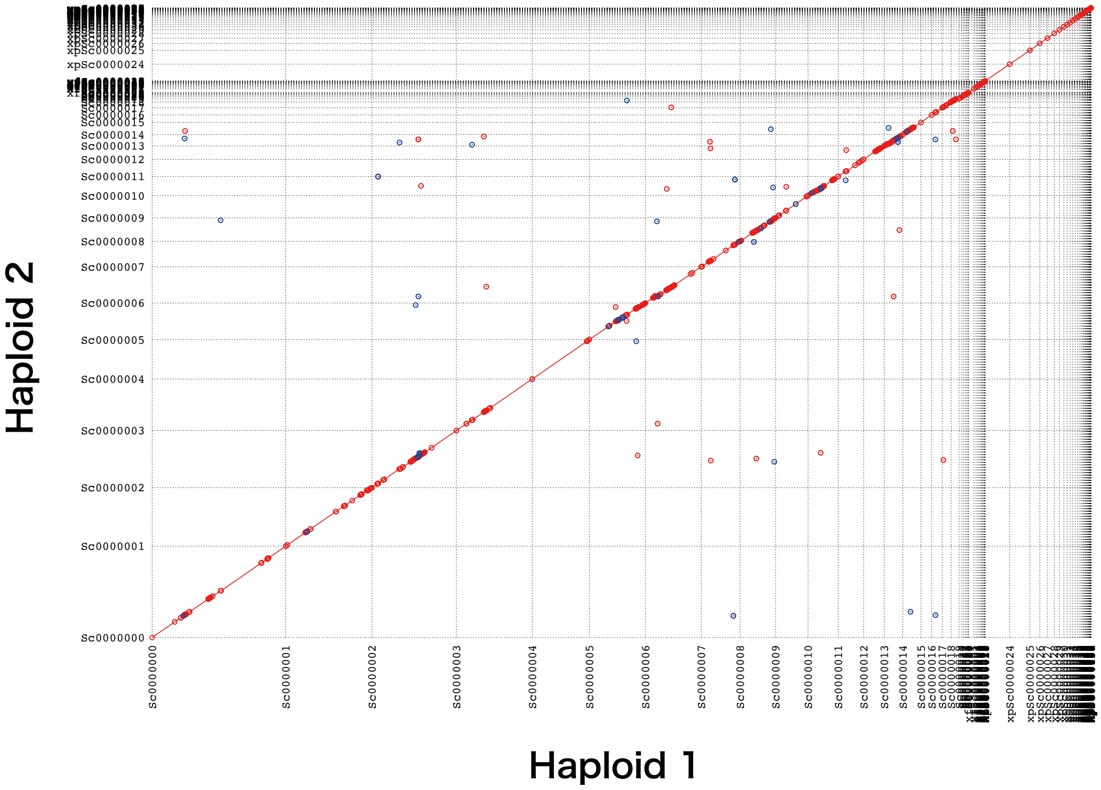

Supplementary Figure 2. Dot plot comparing two haploid sequences. Red and blue plots indicated forward and reverse matches, respectively. This dot plot was left only the alignments which form the longest mutually consistent set.

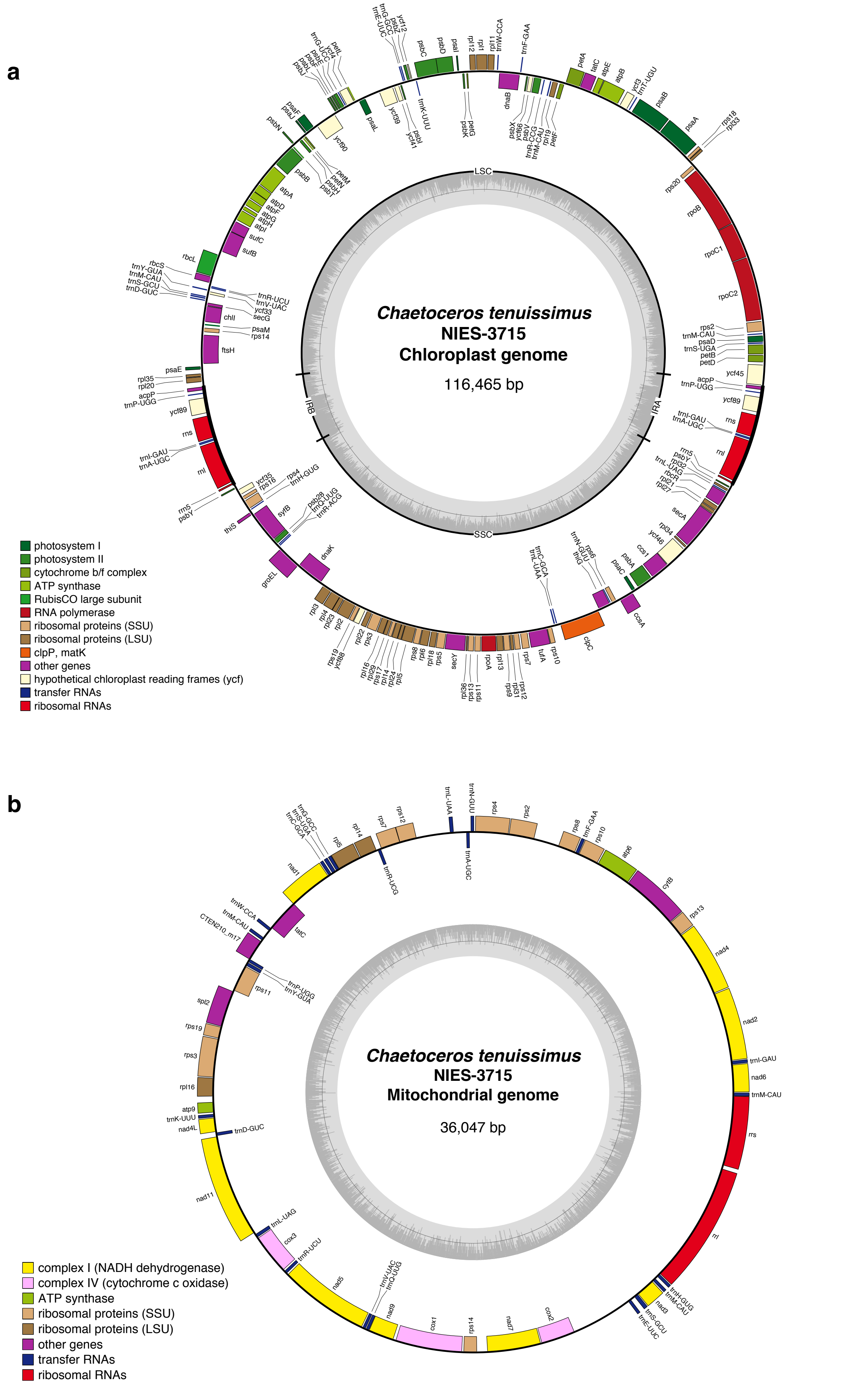

Supplementary Figure 3. Genome map of (a) chloroplast and (b) mitochondrion in *C. tenuissimus* NIES-3715. These maps were drawn by GeSeq annotation server (Reference No.62 in the manuscript).

a

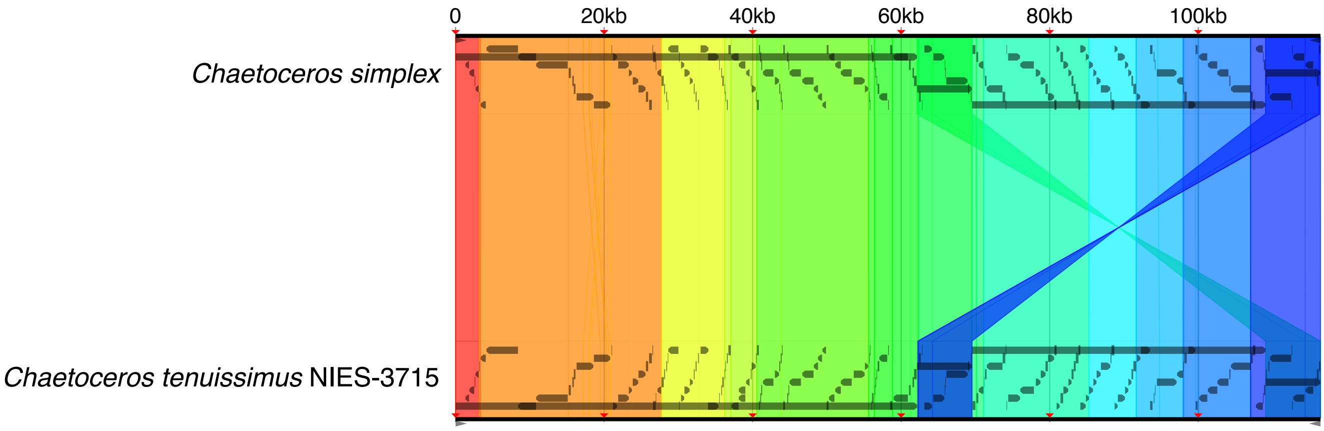

b

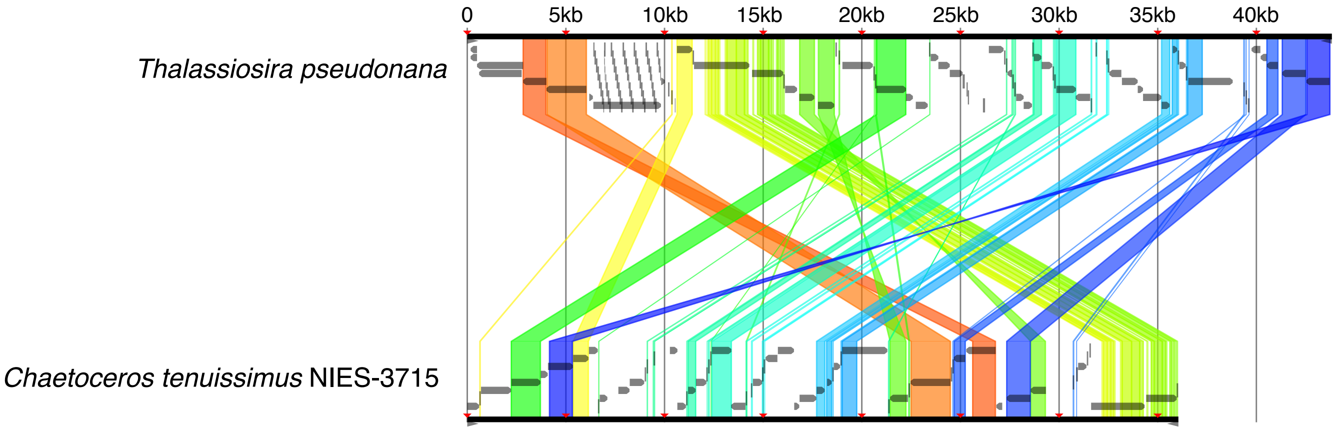

Supplementary Figure 4. Identification of synteny blocks within (a) the chloroplast genome of *C. simplex* and *C. tenuissimus* NIES-3715, and (b) the mitochondrial genome of *T. pseudonana* and *C. tenuissimus* NIES-3715.

```
ctEVLFout_v1_F
cgatcctcttgaagacccagtagtaatgaatgaagatggttgggaccaggtatatactaatttgatattctacctgaatagaaagtattc
ctactaagattctttaccatatactcgcaatctttcaaaatgtcttcaaatattagttgacagagaatttggaatgcgtgtgtagtgagt
aaagagccgttatcatacacaacaagtttgaaatactaacaaatctaacttaatatatcagtaacaacatctgcaactaaggaattattc
ttttcaacatctaaaatgggtagagtagtctaatttgatttttatctgcttgcatattttgctttcatctacggttatcatattttaac
ggctgtgataaaatggcgccattgtttttggcggcattttttttcaatagatcatggatccacactcgaatccttgacctattcactcta

               TSD
cacaattacgttctCATAAAAaaaggagccaacaaaacagtgtcccaactactatttatcttactgaagagctttcctgtgtttcagatc

                                                                    ctEVLFin_v1_F
            Frame 1                                              ctEVLFin_q_v2_F
ttgactgtgcaatggaacaaaattataaaggtgaccgcactagcgtaagaagaagagtcgactggatcaactccaaacctacagattgga
            M  E  Q  N  Y  K  G  D  R  T  S  V  R  R  R  V  D  W  I  N  S  K  P  T  D  W

                                                                          ctEVLFin_q_v1_F
                                      ctEVLFin_q_v2_P                          ctEVLFin_q_v2_R
cttgggcacagatttgcacgagaacaatgaatctaaatatttgttagctgcttgctcctggggtcgcaagtatcacaagtcacgctctg
T  W  A  Q  I  L  H  E  N  N  E  S  K  Y  L  L  A  A  C  S  W  G  R  K  Y  H  K  S  R  S

                                                                           ctEVLFin_q_v1_P
caagtgctcctcgtagaaccattcaaaatgtcatcttaatctacggtgctccttctaccggtaaaacaactgcagccatagactgggata
A  S  A  P  R  R  T  I  Q  N  V  I  L  I  Y  G  A  P  S  T  G  K  T  T  A  A  I  D  W  D

                                ctEVLFin_q_v1_R
ggcaagacaatgagtctgaaggtgctcgctacttcagacgtaatatggactttggtaaattctggggtggtggaaccgcagcatatcttg
R  Q  D  N  E  S  E  G  A  R  Y  F  R  R  N  M  D  F  G  K  F  W  G  G  G  T  A  A  Y  L
gtcaacgcattatccattatgaggaattcaatggaaaagaagaatttcataaatttaaggaaatctgtgatatcggacaagttgggccac
G  Q  R  I  I  H  Y  E  E  F  N  G  K  E  E  F  H  K  F  K  E  I  C  D  I  G  Q  V  G  P

                                     ctEVLFin_v1_R
gtgttaaaatcaaaggttcgagtatcgagctcaatcatgagaccgttattgtgacctctcatgttcacccagcaggctggtatgactctt
R  V  K  I  K  G  S  S  I  E  L  N  H  E  T  V  I  V  T  S  H  V  H  P  A  G  W  Y  D  S
      Insertion
              Frame 2
attggaagggttactgaagttagatttacccagctacaagaccagatggctcgcaaaattatcctaacgaatccaatccaccttacttc
Y  W  K   V  T  E  V  R  F  Y  P  A  T  R  P  D  G  S  Q  N  Y  P  N  E  S  N  P  P  Y  F

atcaatcagactgaggattggaaaaacataatgggtgactaccagtcctgtctagaccattctcagtaacattggccattataattggac
 I  N  Q  T  E  D  W  K  N  I  M  G  D  Y  Q  S  C  L  D  H  S  Q  -  H  W  P  L  -  L  D
    Insertion
          Frame 1                                              poly A-like sequence
gaaggtccccctcgtccaaggggatttgattggacttttgtcaccagtgcagtacaattttgaaataaaaaagaacaaacattgttgcgta
 E  G   P  R  P  R  G  F  D  W  T  F  V  T  S  A  V  Q  F  -

                       TSD
tcgagttcaagtaatattgttcCATAAAAtagcaacacaaagttatactgtgaaagtgatattattttctttatctcaatttatacagtc

                                                           ctEVLFout_v1_R
tctcccatcatatctaaaatatacctattgataatatctttctccgatatatcaacaaacgtgtttgc
```

Supplementary Figure 5. Detail of EVLF sequence information in Figure 1a.

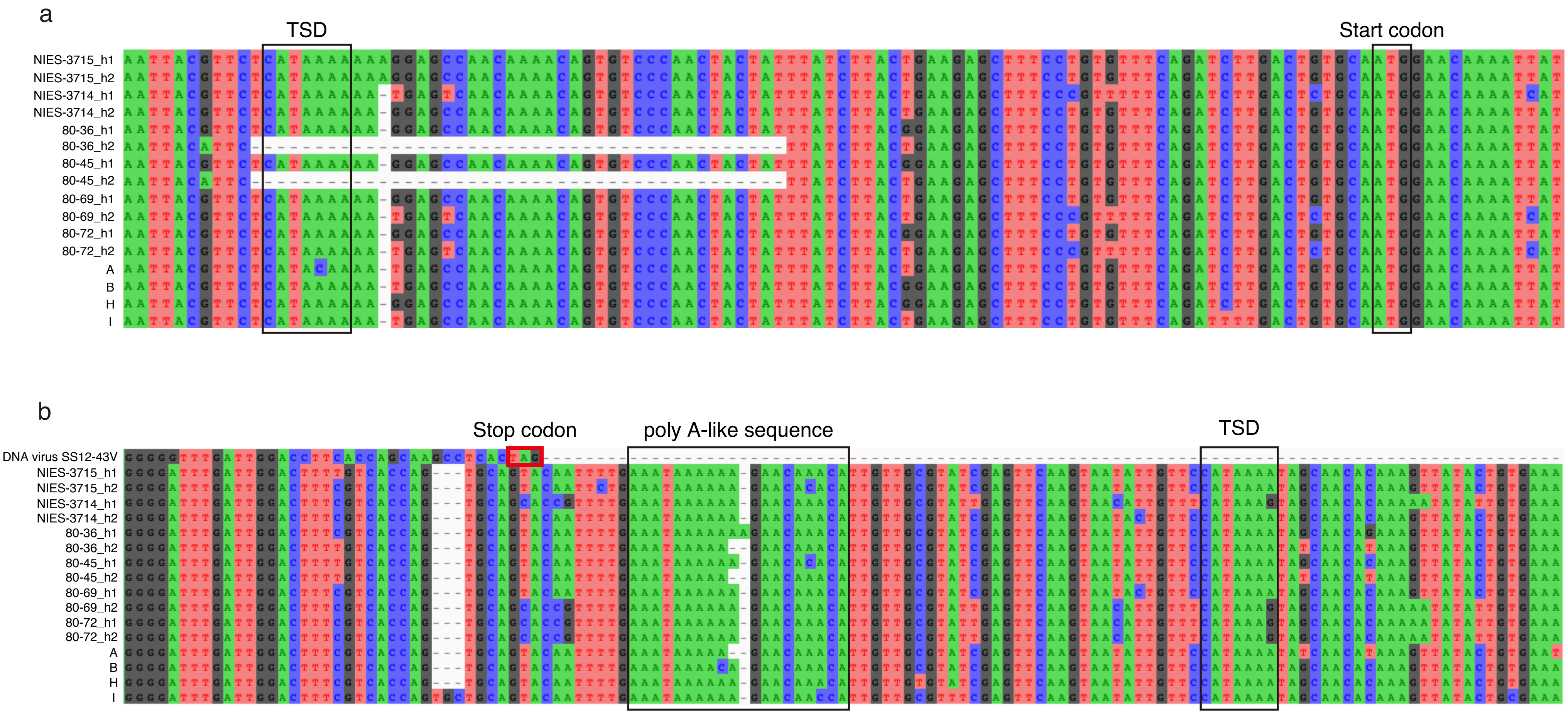

Supplementary Figure 6. Characteristic of EVLF sequence integrated by LINE at (a) 5’ and (b) 3’ -termination.

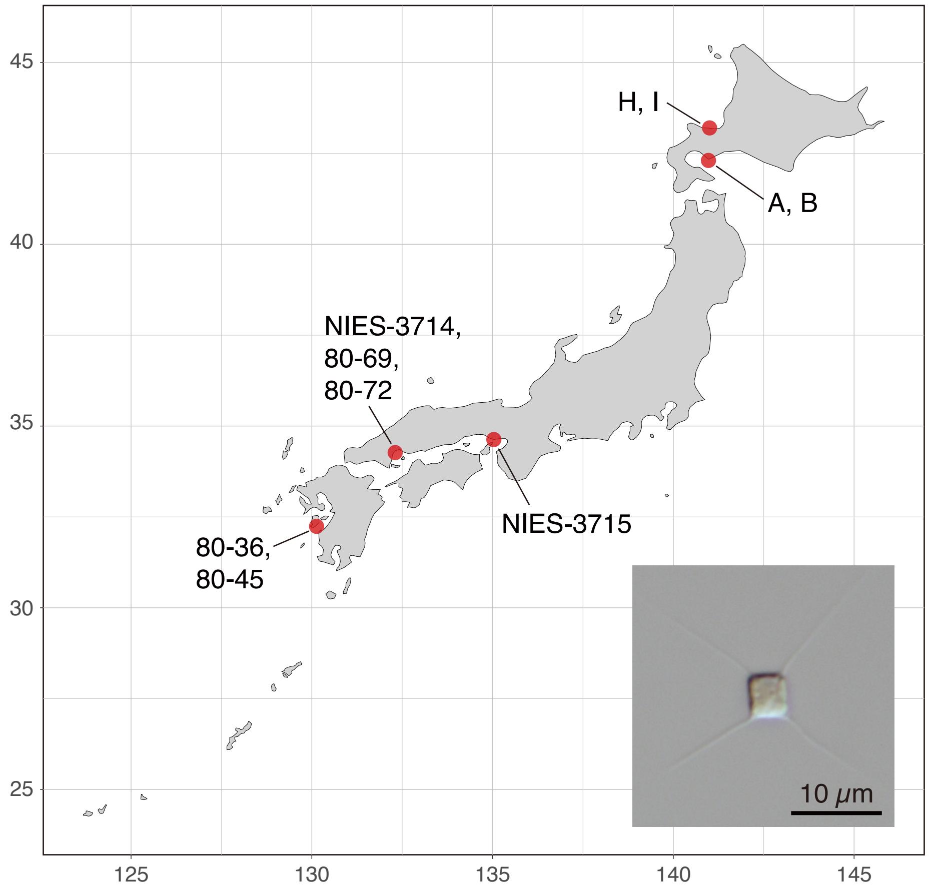

Supplementary Figure 7. Geographic location of the isolated *C. tenuissimus* strains and photographic image of a *C. tenuissimus* cell. *C. tenuissimus* strains: NIES-3714, NIES-3715, A, B, H, I, 80-36, 80-45, 80-69, and 80-72.
